# Resistance is futile: overcoming resistance to targeted therapies in lung adenocarcinoma

**DOI:** 10.1038/s41698-017-0007-0

**Published:** 2017-03-20

**Authors:** Dana S. Neel, Trever G. Bivona

**Affiliations:** 1 Department of Medicine, University of California at San Francisco, San Francisco, CA USA; 2 Department of Cellular and Molecular Pharmacology, University of California at San Francisco, San Francisco, CA USA; 3 Helen Diller Family Comprehensive Cancer Center, University of California at San Francisco, San Francisco, CA USA

## Abstract

The advent of genomics has led to the identification of specific “driver” mutations in oncogenic kinases, and the development of targeted small molecule inhibitors to block their tumor-driving functions. These specific inhibitors have been a clinical success, and often significantly prolong the lives of individuals with cancer. Inevitably, however, the treated tumors recur as resistance to these targeted therapies develops. Here, we review the major mechanisms by which a cancer cell can evade targeted therapy, focusing on mechanisms of resistance to kinase inhibitors in lung cancer. We discuss the promising concept of rational upfront polytherapy in lung cancer, which involves concurrently targeting multiple proteins in critical signaling pathways in a cancer cell to prevent or delay resistance.

## Targeting driver oncogenes in lung adenocarcinoma

A significant fraction of lung adenocarcinomas harbor activating-mutations in targetable oncogenes. These include mutations in *EGFR* (~11%) and *BRAF* (~7%), and activating gene rearrangements involving *ALK* and *ROS1* (1–2%), all of which encode protein kinases, and result in hyperactivation of downstream signaling pathways that drive cell growth, proliferation, and survival.

The identification of these driver kinases has led to the clinical use of small molecule kinase inhibitors that suppress these oncoproteins—erlotinib, gefinitib, afatinib, osimertinib for mutant EGFR, vemurafenib and dabrafenib for mutant BRAF, and crizotinib, ceritinib, alectinib for ALK and/or ROS1 gene rearrangements.^1–10^ These targeted drugs function as ATP-competitive inhibitors. Additionally, inhibitors of kinases that are activated downstream of these oncoproteins have been developed for use as either monotherapy, or in combination with inhibitors of the upstream oncoprotein. The MEK1/2 inhibitor trametinib is one such drug—it inhibits MAPK pathway activation by binding to and blocking MEK in an allosteric fashion. All of these inhibitors have shown efficacy over conventional chemotherapies in patients harboring the cognate genetic driver kinase.

## Mechanisms of resistance to targeted therapies

**Fig. 1:**
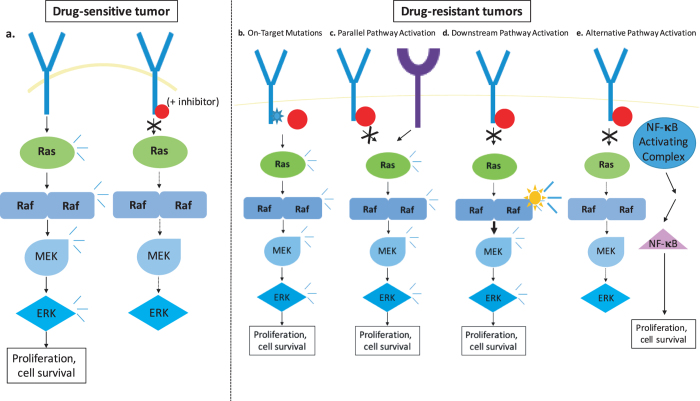
Mechanisms of resistance to targeted therapies. **a**. Example of a drug-sensitive tumor. Downstream signaling is decreased upon addition of a targeted inhibitor. **b**–**e**. Examples of mechanisms promoting drug-resistant tumors. **b**. On-target mutations block the ability of the drug to bind to and inhibit the target oncoprotein, allowing continued signaling to promote tumor survival. **c**. Upregulation of a distinct receptor tyrosine kinase sustains signaling through a critical signaling pathway despite continued inhibition of the primary oncoprotein with the targeted drug. **d**. Mutational activation of a protein involved in a critical downstream signaling pathway reactivates the pathway below the level of inhibitor blockade. **e**. Activation of pro-survival signaling networks can prevent inhibitor-mediated apoptosis

Unfortunately, the initial clinical response to targeted kinase inhibitors is almost always temporary, as acquired resistance to these drugs invariably develops. Many mechanisms of resistance to each targeted therapy have been identified, but can be generally categorized into three predominant classes (Fig. 1): (1) those that alter the driver oncogene, (2) those that activate a critical signaling pathway(s) in a parallel or downstream fashion, and (3) those that drive pro-survival signaling through a different signaling pathway. A fourth class of resistance encompasses histological transformation from one cell lineage such as epithelial to another such as neuroendocrine or mesenchymal. This last class is generally poorly understood.

### Alteration of the driver oncogene

#### Gatekeeper mutations and other “on-target” mechanisms of resistance

Small molecule kinase inhibitors bind to their target through non-covalent bonds within the ATP-binding pocket. Cancer cells can develop resistance to specific small molecule kinase inhibitors by mutating a so-called “gatekeeper” residue within the pocket. This residue is often small in the native oncoprotein with the secondary resistance-associated mutation resulting in a bulky amino acid substitution. How gatekeeper mutations cause resistance to small molecule inhibitors remains incompletely understood. Initial studies showed that the gatekeeper mutation both creates a residue that cannot a hydrogen bond with the inhibitor, and sterically hinders inhibitor binding in the pocket, while leaving the pocket’s ATP-binding affinity unchanged.^11^ The effect of the gatekeeper amino acid substitution is to prevent kinase-inhibitor binding while allowing retention of the ability of the kinase to bind ATP. More recently, data demonstrating that gatekeeper mutants can retain sensitivity to structurally similar but irreversible inhibitors suggest that steric hindrance may not explain the gatekeeper mechanism of resistance in all cases, and instead that the function of a gatekeeper mutation could be to bind ATP more strongly to decrease the ability of the ATP-competitive kinase inhibitor to bind in the pocket.^12^ Which of these mechanisms is responsible for gatekeeper mutation-mediated inhibitor resistance may depend on the kinase in question.

The gatekeeper T790M mutation in EGFR is found in ~50% of EGFR-mutant patients who develop resistance to EGFR inhibition.^13, 14^ Gatekeeper mutations have similarly been identified in cancers that become resistant to ALK inhibitors (L1196M). Additionally, other “on-target” mutations in EGFR and ALK have been described, which are found at other residues within the ATP-binding domain and cause targeted inhibitor resistance.^15–17^ While the gatekeeper threonine mutation has been identified as an in vitro cause of BRAF inhibitor resistance, this mutation has not been seen in patients.^18^ This may be because many BRAF inhibitors induce paradoxical activation of wild-type RAF.^19^ Thus, upregulation of wild-type RAF or RAS signaling can activate the same pathways that are downstream of mutant BRAF. These signaling events may obviate the selective pressure to acquire resistance to so called “paradox activating” RAF inhibitors by mutation of the driver oncogene itself.

The prevalence of on-target mutations as a mechanism of resistance has led to the creation of new inhibitors that can inhibit both the original oncoprotein and its resistance-associated mutated form. While this strategy does improve patient survival in the short term, mechanisms of resistance emerge to these inhibitors as well.^4, 20^

Other alterations in the driver oncogene can lead to resistance as well. Studying BRAF^V600E^ mutant lung adenocarcinoma, our group identified that a switch from expression of a full-length BRAF^V600E^ to a shorter splice variant was capable of mediating resistance to the BRAF inhibitor vemurafenib.^21^ This splice variant is able to dimerize and activate downstream MAPK pathway signaling despite presence of the RAF inhibitor. This mechanism of resistance was observed initially in BRAF-mutant melanomas, indicating conservation across different tumor histologies.^22^

Finally, changes in the level of the targeted driver oncogene can also cause inhibitor resistance. By increasing the levels of the driver oncogene, an inhibitor appears to be less potent, because there will be fewer molecules of inhibitor per molecule of oncoprotein. Upregulation of BRAF^V600E^ was identified as a driver of resistance in inhibitor-insensitive melanomas.^23^ Intriguingly, loss of the initial driver oncogene and replacement with a new driver, also known as “oncogene swap”, has recently been described as a cause of targeted inhibitor resistance.^24^ In this instance, a cancer cell loses the targeted driver oncogene, and upregulates a new oncogene, such that oncogenic signaling driving cell survival is mediated by the new oncogene, and the inhibitor of the initial oncogene no longer has a substantial impact on cancer cell survival.

### Reactivation of critical signaling pathways

#### Parallel activation of signaling pathways

One way that cancer cells can evolve resistance to a targeted therapy without altering the target oncogene is by upregulating expression and/or activation of a protein that signals through the same signaling pathway. In this way, despite continued suppression of the initial driver oncoprotein by the small molecule inhibitor, critical parallel signaling persists under the control of the newly upregulated protein activity.

Amplification of wildtype *MET* in the setting of EGFR mutant lung cancer was one of the earliest examples of this type of mechanism of resistance.^25–27^ Mutant EGFR heterodimerizes with ErbB-3 to activate the PI3K/Akt signaling pathway. Upon inhibition of EGFR with a targeted inhibitor, this pathway is suppressed. *MET* amplification leads to the reactivation of this pathway by forming a MET-ErbB-3 heterodimer.^28^ Thus, despite continuous suppression of EGFR by the inhibitor, a critical downstream signaling pathway is reactivated, and resistance to the inhibitor emerges. Upregulated expression of the MET ligand HGF, leading to hyperactivation of MET, has been found to drive resistance to EGFR inhibition through a similar mechanism as *MET* amplification.^29, 30^ Clinical trials are underway to test the effect of dual inhibition of MET and EGFR to overcome this mode of resistance.^31^ Additionally, ErbB-3 blocking antibodies are currently under clinical development.^32^ Amplification of the ERBB family member *HER2* has also been identified as a possible mechanism of resistance to EGFR inhibitors by similarly activating ErbB3 and downstream PI3K signaling.^33^ Inhibition of this key signaling pathway downstream of the activation node is another way to overcome resistance.^34^

Upregulation or activation of non-ERBB family member receptor tyrosine kinases to reactivate downstream signaling pathways, and cause resistance has also been identified. IGF-1R pathway activation can mediate resistance in both *EGFR*-mutant and *EML4-ALK* positive lung cancer.^35–37^ Overexpression of the tyrosine kinase AXL, which is able to engage multiple downstream signaling nodes also activated by EGFR, was identified by our group and others as a driver of EGFR inhibitor resistance.^38^ Similar findings were observed in ALK gene rearrangement positive lung adenocarcinoma.^39^

Similarly, resistance to Raf inhibition can result from upregulated levels of EGF, which activates EGFR, and drives downstream pathway activation in BRAF^V600E^ mutant lung adenocarcinoma.^21^ Combinatorial inhibition of multiple kinases could be a therapeutic possibility in cases where activation of alternative receptor tyrosine kinases is responsible for driving resistance.

#### Downstream activation of signaling pathways

Cancer cells can become resistant to targeted inhibitors by amplifying or acquiring activating mutations in pathway genes downstream of the driver oncogene. Thus, despite continued silencing of an oncoprotein’s activity by its inhibitor, there is persistent pathway activation downstream of the reactivated pathway node.

Activating mutations in or amplification of genes involved in MAP kinase pathway signaling have been identified as a mechanism of resistance to targeted inhibitors of several driver oncogenes. *BRAF* mutations drive resistance to targeted inhibition of EGFR, while *KRAS* amplification leads to ALK kinase inhibitor resistance in EML4-ALK-drive lung cancer.^40, 41^ Similarly, downregulation of genes that negatively regulate the MAP kinase pathway have also been implicated in resistance. Decreased expression of the phosphatase *DUSP6* leading to rescue of phospho-ERK and reactivation of MAPK signaling was observed in ALK inhibitor-resistant cell lines and patient samples.^40^ Similarly, downregulation of *NF1*, leading to reduced levels of the Ras-GTPase activating protein neurofibromin, and thus increased Ras activity, has been identified as a driver of resistance to EGFR inhibition in lung cancer.^42^

Along with signaling through MAPK, the PI3K pathway is another major signaling pathway that is commonly hyperactivated downstream of driver oncogenes. Several lines of evidence suggest that downstream reactivation of this pathway may be a mechanism of acquired resistance to targeted inhibitors. First, concurrent *EGFR* and *PIK3CA* mutations are seen in patients with acquired resistance to EGFR inhibition, and are correlated with a poor response to EGFR-targeted therapy; furthermore, PIK3CA mutants can drive resistance to EGFR inhibitor in vitro.^43, 44^ These mutations lead to increased phosphorylation of PIP2, generating the second messenger PIP3 and activating AKT. Similarly, homozygous loss of *PTEN*, which normally acts to dephosphorylate PIP3 and suppress this signaling pathway, has also been identified as a mechanism of EGFR inhibitor resistance in vitro in lung and other cancers.^45, 46^ However, the clinical significance of these mutations with regard to inhibitor sensitivity remains controversial. While concurrent mutations in the PI3K pathway with EGFR and KRAS mutations are associated with a poorer prognosis overall, PI3K mutations do not alter sensitivity or clinical responses to targeted inhibitors in EGFR-mutant lung cancer, suggesting reactivation of this pathway may not actually be a cause of targeted inhibitor resistance in patients.^47^

### Alternative pathway activation

#### Activation of pro-survival signaling pathways

Besides reactivation of critical pro-growth pathways downstream of a driver oncogene, activation of pro-survival signaling networks has also been implicated in targeted inhibitor resistance. Our group discovered that, upon inhibition of EGFR, an NFκB-containing complex is rapidly recruited, and activates a downstream pro-survival signaling pathway that limits EGFR inhibitor-mediated cell death.^48, 49^ Co-treatment of cells with EGFR and NFκB inhibitors prevents the emergence of this resistance in pre-clinical models.^49^

Activation of pro-survival genes also limits response to MAPK pathway inhibitors in BRAF and RAS-driven tumors. Our group and others identified the YAP pathway as a key mediator of response to MAPK pathway inhibition in these tumors.^50–52^ In some tumors, YAP levels are elevated and drive transcription of downstream targets, including *BCL2L1*, which encodes the anti-apoptotic BCL-xL protein. These tumors are more resistant to MAPK pathway inhibitors because, even without pro-survival MAPK pathway signaling, these tumors maintain activation of alternative pro-survival signaling pathways through YAP activation.

These mechanisms of resistance are distinct from those discussed earlier, as they involve engagement of pathways separate from those activated by the driver oncogene prior to inhibitor treatment. In both cases described here, combined inhibition of the driving oncogenic pathway and the resistance-mediating pro-survival pathway is an effective way to overcome targeted inhibitor resistance.^49, 50^

### Additional mechanisms of resistance

#### Epigenetically regulated drug tolerance

Global epigenetic changes have been observed in response to small molecule inhibitor treatment. Sharma and colleagues found that the H3K4 histone demethylase KDM5A was upregulated in inhibitor-resistant EGFR-mutant cells, and this overexpression was required for drug resistance.^53^ Intriguingly, IGF-1R signaling was found to be required for establishment of this drug-resistant cell population, and *KDM5A* upregulation was linked to IGF-1R signaling. This finding suggests that upregulation of IGF-1R could contribute to inhibitor resistance through multiple mechanisms, both by reactivating critical signaling pathways (as discussed earlier) and by altering a cell’s epigenetic landscape.

#### Germline disruption of apoptosis

Germline polymorphisms in pro-survival signaling pathways have also been implicated in targeted inhibitor resistance. Ng and colleagues identified a common polymorphism in *BIM*, a pro-apoptotic protein, as a mediator of intrinsic targeted inhibitor resistance in EGFR-driven lung cancer and chronic myeloid leukemia.^54^ Normally, MAP kinase pathway signaling activated by the driver oncogene suppresses BIM and it’s pro-apoptotic function. Upon treatment with a targeted inhibitor, this suppression is released, restoring the pro-death function of BIM. However, some patients harbor a polymorphism in *BIM* that lacks the critical pro-apoptotic BH3 domain, thus rendering BIM ineffective in driving apoptosis upon oncogene inhibition and causing intrinsic resistance to the targeted inhibitor. In these cases, Ng *et al.* suggest BH3 mimetics in combination with targeted oncogene inhibition may be efficacious in combating intrinsic resistance.

#### Upregulation of drug transporters

One non-specific mechanism of small molecule inhibitor resistance is upregulation of drug efflux pumps. Expression of these pumps has long been associated with resistance to a variety of cytotoxic cancer therapies, as they reduce intracellular drug concentration. Some small molecule tyrosine kinase inhibitors are targets of efflux pumps, and in some cases, expression of specific drug transporters have been reported to induce drug resistance to targeted inhibitors of EGFR and ALK.^55, 56^

#### Epithelial-to-mesenchymal transition (EMT)

The EMT has been observed in association with acquired resistance to targeted inhibitors in a variety of oncogene-driven cancers, including EML4-ALK and EGFR mutant lung adenocarcinoma.^38^ EMT is observed as a morphologic change in cells, decreased epithelial markers (like E-cadherin) and upregulated mesenchymal markers (like vimentin), as well as increased migration and invasion properties of cells. However, whether EMT is directly responsible for acquired resistance or is an associated but not causal change remains controversial. For example, induction of EMT by TGFB signaling is associated with ALK inhibitor resistance in an EML4-ALK-positive cell line, and knockdown of the mesenchymal marker vimentin restores sensitivity in an EML4-ALK-acquired resistance model that displays EMT.^57^ However, other groups have found that reversal of the EMT phenotype in ALK inhibitor-resistant EML4-ALK lines can be induced despite continued resistance to the targeted inhibitor.^58^ Thus, whether or not EMT is a driver of resistance or just a consequence associated with the functional resistance mechanism remains to be elucidated.

#### Transformation to small cell carcinoma

Finally, histologic transformation of non-small cell lung adenocarcinoma to small cell carcinoma is occasionally observed as a mechanism of resistance to multiple driver oncogene-targeted therapies, including inhibitors of EGFR and EML4-ALK.^59–61^ Interestingly, these tumors retain expression of the original mutant oncogene, and do not appear to have acquired any of the resistance mechanisms discussed above. Analysis of these small-cell-transformed tumors revealed loss of *RB1*, a hallmark of small cell lung cancer.^62, 63^ Loss of *RB1* may be the alteration that induces this non-small-cell to small-cell transformation, resulting in a molecular switch from dependence on the original driver oncogene to a different survival program regulated by *RB1* loss.^64^

### Emergence of resistance

Most patients will have an incomplete response to a given targeted therapy—that is, their tumors will not completely regress, and they will have some degree of residual disease from which acquired resistance subsequently emerges.^10^ While much research over the past decade has focused on identifying mechanisms mediating acquired resistance to targeted inhibitors, there is still much to be learned about what other factors might be involved in preventing an initial complete response and supporting residual disease. This population of tumor cells that never completely responds to therapy likely functions as a transition state culminating eventually in a drug-resistant tumor (acquired resistance). Therefore, understanding the mechanisms involved in the survival of this subpopulation of residual tumor cells is critical for progress.

How resistance emerges in a tumor remains unclear. One possibility is that certain resistance mechanisms exist in a subset of tumor cells prior to treatment with a targeted agent. These pre-existing resistant cells are then selected for upon introduction of the targeted inhibitor. Many tumors show a great amount of intratumoral heterogeneity prior to therapy, and there is evidence of the existence of tumor cells harboring resistance mutations prior to the treatment with targeted inhibitors.^65–68^ Another possibility is that the treatment of tumor cells with a targeted inhibitor induces a drug-tolerant population of cells from which resistance can then emerge based on the stochastic presence of genetic variation due to the underlying mutation rate in replicating cells.^53, 68^ A third hypothesis is that targeted inhibition of an oncogene itself somehow induces epigenetic or genetic changes, leading to resistance. Likely, any or all modes of resistance emergence may occur in an individual patient, depending on the intratumoral heterogeneity present prior to treatment and the characteristics of the cancer type and inhibitor used. However, if critical signaling pathways can be predicted and targeted in a patient in an upfront manner, the emergence of resistance through either mechanism described here may be delayed or prevented entirely, offering a promising approach to combat the heterogeneity and adaptiveness of most cancers.

## Development of rational upfront polytherapy strategies

Despite initial frustrations over the inevitable emergence of resistance to targeted kinase inhibitors, new insights into understanding these mechanisms of resistance provide hope for the development of more durably effective treatment strategies. The emergence of resistance can suggest which pathway(s) are critical for the survival of cells driven by specific oncoproteins, suggesting the potential of upfront combinatorial inhibition of the driver oncoprotein and the crucial pathway further downstream. Preclinical studies from our group have demonstrated that upfront treatment of EML4-ALK positive lung tumors with both an ALK inhibitor and a MAPK pathway inhibitor can significantly delay or even prevent onset of resistance.^40^ The development of clinical trials to test this upfront therapy in patients is underway. Similar findings were observed in EGFR mutant lung adenocarcinoma preclinical models and individuals with BRAF V600E lung adenocarcinoma.^69, 70^

So far in this review, we have covered the myriad ways a cancer cell can develop resistance to targeted therapies. But how can the knowledge of how cancer cells evade inhibitors translate into real benefits for patients? Now that many pathways of resistance have been identified, these specific resistance mechanisms can be monitored for in the patient once they start inhibitor treatment. In many cases, additional drugs are available that can be combined with the initial inhibitor to overcome emerging resistance. The fact that tumor cells shed their DNA, which can be detected in the blood of patients, has recently begun to be harnessed to effectively obtain serial biopsies of a patient’s disease over the course of treatment. In this way, physicians can easily monitor for the emergence of genetic alterations associated with resistance to inhibitors, and potentially deploy combination therapies to short-circuit the emergence of resistance before it is fully established in the patient.^71, 72^ Early pre-clinical and clinical data suggest it may be critical to combat resistance by treating it before it truly emerges—once a tumor has recurred on a macroscopic level, it appears to be less sensitive to inhibitors targeting its resistance mechanism as when a tumor is initially co-treated with inhibitors targeting both its driver oncogene and a common resistance mechanism.^73, 74^ This clinical experience exemplifies the importance of early low-level detection of residual disease and tumor recurrence using liquid biopsies (and where feasible on-treatment tumor biopsies) so resistance can be addressed prior to the appearance of macroscopic disease, and of understanding the signaling pathways that are most critical to tumor survival to allow for upfront polytherapy to prevent resistance from ever appearing.^10^

While this review mostly focuses on resistance to targeted therapies in lung cancer, the mechanisms can be broadly applicable to other tumor types as well. First, many other tumors that harbor the same driver oncogenes discussed here, are targetable with the same small molecule inhibitors, and show similar patterns of resistance mechanisms. Second, identification of resistance mechanisms that may emerge, and then upfront treatment of patients with combinations of inhibitors to delay or prevent this resistance from occurring is a concept that is generalizable to virtually any tumor type. Better understanding of how the tumor cells escape from the detrimental effects of targeted therapies holds promise for our ability to prioritize, and deploy synergistic drug combinations that chronically control and potentially eliminate the ability of the tumor to evolve full resistance. While drug combinations may show clinical toxicity, prioritizing combinations of agents with synergistic anti-tumor effects could offer a therapeutic window. Alternatively, sequential or alternating drug schedules could be tested, where appropriate, to mitigate clinical toxicity and maintain anti-tumor efficacy.

Altogether, the path to chronic cancer control is clearer and brighter due to the increasing understanding of the biological basis of resistance, and the arrival and adoption of emerging technologies that allow us to chart the molecular course of cancer evolution in individual patients during treatment.
